# A Case of Progressive Multifocal Leukoencephalopathy Caused by Epcoritamab

**DOI:** 10.7759/cureus.71655

**Published:** 2024-10-16

**Authors:** Chifumi Iseki, Kazuo Nakamichi, Kenichi Ishizawa, Yasuyuki Ohta, Tomomi Toubai

**Affiliations:** 1 Division of Neurology and Clinical Neuroscience, Department of Internal Medicine Ⅲ, Yamagata University, Yamagata, JPN; 2 Department of Behavioral Neurology and Cognitive Neuroscience, Tohoku University Graduate School of Medicine, Sendai, JPN; 3 Department of Virology 1, National Institute of Infectious Diseases, Tokyo, JPN; 4 Department of Nursing, Faculty of Health Sciences, Tohoku Fukushi University, Sendai, JPN; 5 Division of Hematology and Cell Therapy, Department of Internal Medicine Ⅲ, Yamagata University, Yamagata, JPN

**Keywords:** anti-cd-20, follicular lymphoma, lymphocytopenia, progressive multifocal leukoencephalopathy (pml), subcortical

## Abstract

A female patient aged in her 50s had presented with the onset of follicular lymphoma (FL) with left mandibular swelling, with a pathological grade of 1 and clinical stage of Ⅳ (Ann Arbor staging). Cyclophosphamide, doxorubicin, vincristine, prednisolone, and rituximab (R-CHOP) resulted in complete molecular remission (CMR). The patient experienced two recurrences, and treatments were successful; however, the side effect of continuous lymphocytopenia existed eight years after the onset. For the third recurrence of FL, weekly epcoritamab therapy was administered with a white blood cell count of 2,010 /μL with neutrophils of 1,240/μL, lymphocytes of 430/μL, red blood cells of 390 × 10^4^/μL, and platelets of 17.8 × 10^4^/μL. ^18^Fludeoxyglucose positron emission tomography (FDG-PET) confirmed CMR after six cycles of epcoritamab. After the 11th epcoritamab, the patient was diagnosed with progressive multifocal leukoencephalopathy (PML), presenting significant left hemispatial neglect and visuospatial problems. Brain magnetic resonance imaging of fluid-attenuated inversion recovery and diffusion-weighted imaging showed high intensity in the right parietotemporal subcortex and frontal subcortical lesion with high or iso intensity on the apparent diffusion coefficient. FDG-PET did not show lymphoma recurrence. The patient had white blood cells of 2,310 /μL with lymphocytes of 480/μL, CD4-positive lymphocytes of 124/μL, and CD8-positive lymphocytes of 153/μL. The JC virus (JCV) deoxyribonucleic acid (DNA) level in cerebrospinal fluid (CSF) as examined by polymerase chain reaction (PCR) increased to 1.466 × 10^8 ^copies/mL. The patient became unconscious and died three months after diagnosis of PML. We report the first case of PML as a complication of epcoritamab, a bispeciﬁc antibody targeting CD3 and CD20 that redirects and activates T cells, which is expected to be used for treating FL. PML is a fatal infection of the central nervous system without effective treatment caused by the reactivation of the JCV in immunodeficient hosts. The antibody test for JCV is recommended for patients with multiple sclerosis for an earlier diagnosis, which is not common in other diseases. We should be aware of PML through innovative therapy.

## Introduction

We report the first case of progressive multifocal lymphoma (PML) [1] as a complication of epcoritamab, a bispeciﬁc antibody targeting CD3 and CD20 that redirects and activates T cells and is expected to be used for treating follicular lymphoma [2,3]. PML is a fatal infection of the central nervous system without effective treatment caused by the reactivation of the JC virus (JCV) in immunodeficient hosts [1], and the risk of PML is increasing due to the recent development of immunobiological drugs for refractory diseases [4].

## Case presentation

**Figure 1:**
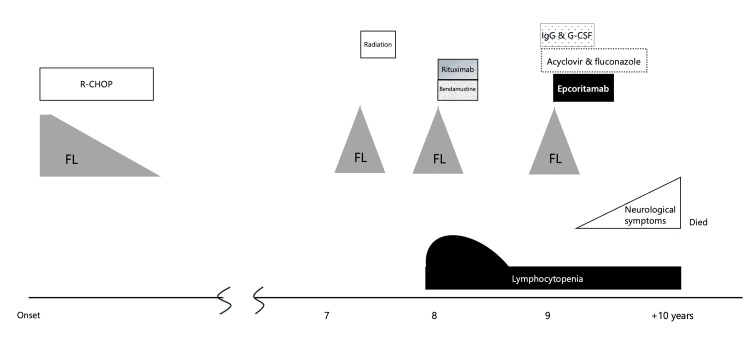
The clinical course over ten years of follicular lymphoma (FL), treatment, and progressive multifocal leukoencephalopathy (PML). The patient had three recurrences of follicular lymphoma (FL) after nine years of the onset. Remission to CMR was achieved by chemotherapy with R-CHOP. The recurrences were treated with radiation, and a combination of rituximab and bendamustine, and epcoritamab. Although these treatments could achieve control of FL; only a course of chemotherapy was used for the second recurrence due to the lymphocytopenia, which recovered partially. The weekly treatment with epcoritamab was stopped after 11 doses due to the development of PML. FL: follicular lymphoma; CMR: complete metabolic remission; R-CHOP: the chemotherapy with cyclophosphamide, doxorubicin, prednisone, rituximab, and vincristine; IgG: immunoglobulin G; G-CSF: granulocyte colony-stimulating factor; PML: progressive multifocal leukoencephalopathy.

**Figure 2:**
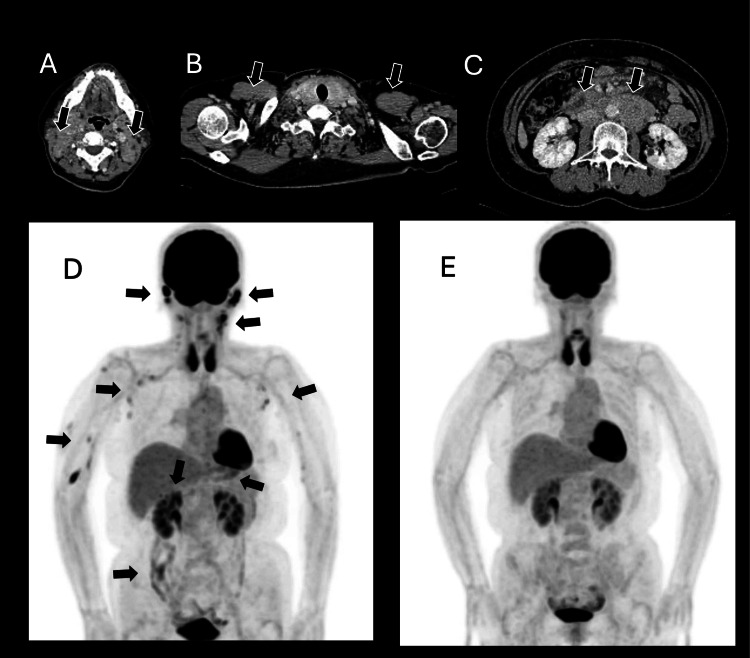
The follicular lymphoma computed tomography (CT) and fluorodeoxyglucose F18 positron emission tomography (FDG-PET). At the onset, the patient had lymph node swellings in the bilateral cervix and larynx ((A) arrows), axilla ((B) arrows), and abdomen ((C) arrows). Nine and a half years after the onset, the lymphoma relapsed. The lymph nodes and organization in the face, neck, parotid glands, and axilla were swollen in FDG-PET ((D) arrows). The FDG-PET confirmed the remission of lymphoma after the sixth injection of epcoritamab (E).

A woman in her 50s presented with left mandibular swelling, which resulted in a diagnosis of follicular lymphoma (FL) of grade 1 and clinical stage Ⅳ of Ann Arbor staging with swelling of lymph nodes in the cervical and mediastinum, thyroid, and spleen (the clinical course is shown in Figure 1) (Figures 2A-2C). The patient received eight courses of chemotherapy with cyclophosphamide, doxorubicin, vincristine, prednisolone, and rituximab (R-CHOP), resulting in complete molecular remission (CMR). Five years later, the patient did not have a significant medical history; however, an aneurysm at the junction of the left internal carotid artery was accidentally found, and clipping was performed. Therefore, the patient was followed up annually via brain magnetic resonance imaging (MRI). Seven years after the onset of FL, radiation therapy for the recurrence of lymphoma at thyroid cartilage brought CMR again. Eight years after FL onset, chemotherapy with rituximab and bendamustine for left eyelid lymphoma recurrence was canceled after only one course because of lymphocytopenia. The lymphocyte count partially recovered until eight and a half years after the onset of FL, although it continued at 200-600/μL. Chemotherapy with epcoritamab at a dose of 0.16 mg was commenced after a recurrence of lymphoma nine and a half years after the onset of FL (Figure 2D). At the start of treatment, the patient’s blood counts were as follows: white blood cells of 2,010 /μL with neutrophils, 1,240/μL; lymphocytes, 430/μL; red blood cells of 390 × 10^4^/μL, and platelet of 17.8 × 10^4^/μL. Immunoglobulin G (IgG), A (IgA), and M (IgM) levels were 537, 13, and 13 mg/dL, respectively. The patient experienced cytokine-releasing syndrome (CRS) of grade 1 with pyrexia but did not have immune effector cell-associated neurotoxicity (ICANS). After the sixth injection, fludeoxyglucose positron emission tomography (FDG-PET) revealed no lymphoma lesions (Figure 2E). Intravenous gammaglobulin therapy and injections of granulocyte colony-stimulating factor maintained this chemotherapy at intervals of at least monthly to several months. Low-dose acyclovir and fluconazole were prescribed to prevent infection.　

**Figure 3:**
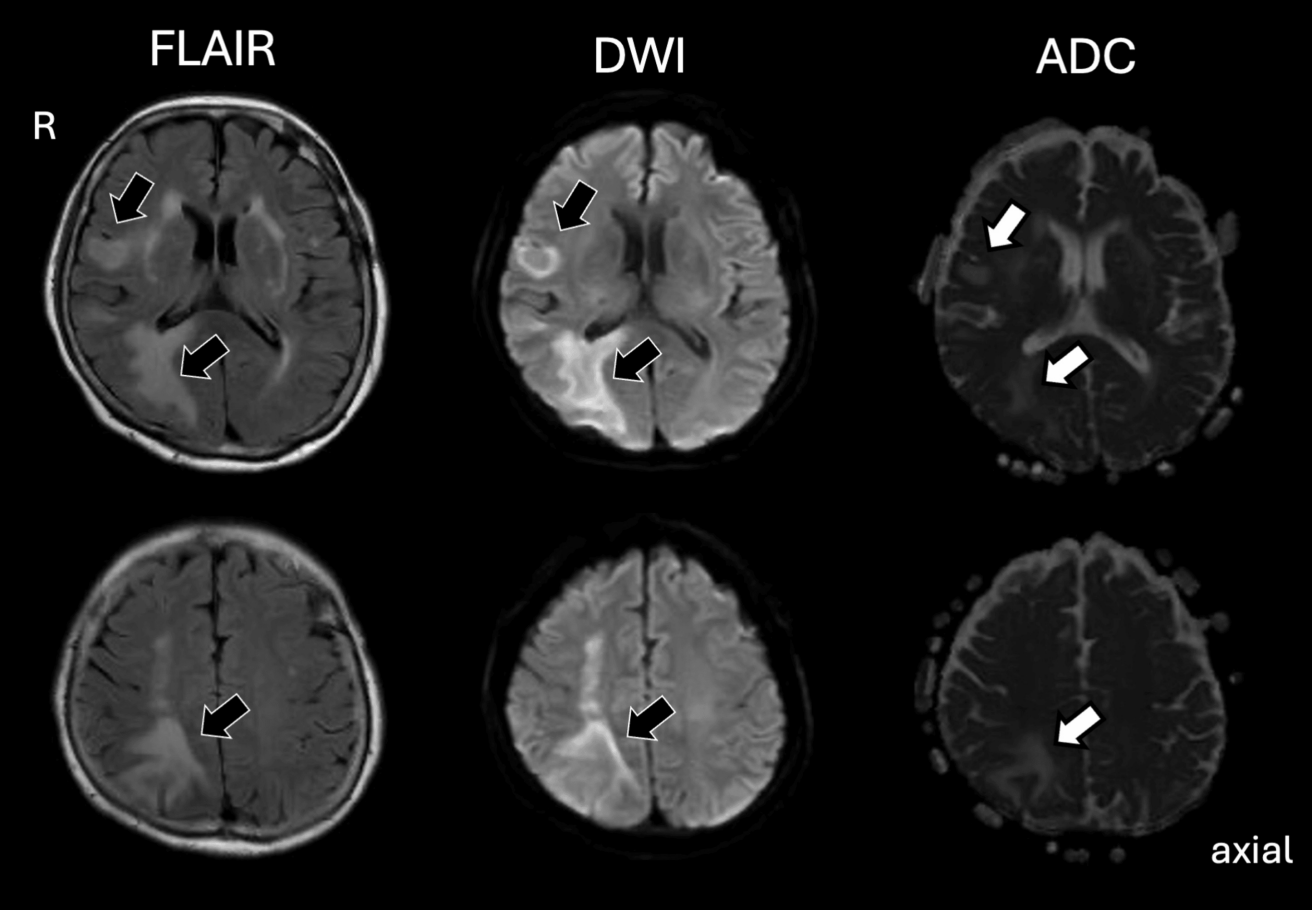
The brain findings of the onset of neurological symptoms of progressive multifocal leukoencephalopathy (PML). Brain MRI with FLAIR sequences and DWI imaging showed high intensity and subtly enlarged lesions in the right parietotemporal and frontal subcortical regions (left and middle). The lesions showed high or iso intensity on apparent diffusion coefficient (ADC) (right). FLAIR: fluid-attenuated inversion recovery; DWI: diffusion-weight image; ADC: apparent diffusion coefficient.

Neurological symptoms developed after the 11th dose of epcoritamab, 48 mg. The patient had trouble seeing and dressing and felt a tingling sensation in the left hand. Neurological examination showed left homonymous hemianopsia, left hemispatial neglect, significant Bálint syndrome (simultanagnosia, oculomotor apraxia, and optic ataxia), construction apraxia, and subtle left-sided hemiplegia without ambulation disturbance. The patient’s mini-mental state examination was 24. Brain MRI with fluid-attenuated inversion recovery (FLAIR) sequence and diffusion-weighted imaging (DWI) revealed intense high signals in the right parietal and temporal subcortices and U-fiber lesions lying beneath the cortices of the frontal lobe (Figure 3, left and middle). The lesions signaled low intensity and partial cortical laminar high intensity on T1-weighted images without gadolinium contrast and high or iso intensity on apparent diffusion coefficient (ADC) (Figure 3, right). These subcortical lesions showed expansion of the affected territories. The characteristics of the lesions were not attributed to lymphoma recurrence. Repeated FDG-PET did not show lymphoma recurrence in the cranium or lymph nodes throughout the body. Further examinations were conducted for diagnosis. Laboratory data obtained at the time which confirmed a diagnosis of lymphocytopenia were as follows (see Table 1): white blood cells of 2,310 /μL with neutrophils, 1,840/μL; lymphocytes, 320/μL; monocytes, 190/μL, eosinophils of <10/μL, basophils, 20/μL, red blood cells of 443 × 10^4^/μL, hemoglobin, 13.3 g/dL, hematocrit, 41.3%, and platelets of 21.5 × 10^4^/μL. IgG, IgA, and IgM were 526, 12, and 9 mg/dL, respectively. A month before the onset of neurological symptoms, the patient’s CD4- and CD8-positive lymphocytes were 124/μL and 153/μL, respectively, when the total lymphocytes were 480/μL. Biochemical data did not show any disorders with total protein of 6.0 g/dL, albumin of 4.1 g/dL, total bilirubin of 0.7 mg/dL, aspartate transaminase of 19 U/L, alanine transaminase of 11 U/L, lactate dehydrogenase of 242 U/L, alkaline phosphatase of 112 U/L, blood urea nitrogen of 12 mg/dL, creatinine 0.60 mg/dL, sodium of 141 mmol/L, potassium 4.2 mmol/L, chloride of 101 mmol/L, calcium of 9.5 mg/dL, glucose 106 mg/dL, C-reactive protein of <0.10 mg/dL, and anti-aquaporin antibody antibodies of <1.5 U/mL.

**Table 1:** The laboratory data at the onset of neurological symptoms. *The data were collected one month before the other data. EBV: Epstein-Barr virus; DNA: deoxyribonucleic acid; HSV: herpes simplex virus; VZV: varicella zoster virus, JCV: JC virus.

Laboratory data	Patient	Normal range	Units
Hematological and blood chemical examinations			
White blood cells	2,310	3,300-8,600	/μL
Neutrophils	1,840	1,700-6,460	/μL
Lymphocytes*	480	970-3,170	/μL
CD4-positive lymphocytes*	124	344-1,289	/μL
CD8-positive lymphocytes*	153	110-1,066	/μL
Monocytes	190	190-660	/μL
Eosinophils	< 10	20-470	/μL
Basophils	20	10-80	/μL
Red blood cells	4.43 × 10^6^	3.86-4.92	/μL
Hemoglobin	13.3	11.6-14.8	g/dL
Hematocrit	41.3	35.1-44.4	%
Platelets	215 × 10^3^	138-348	/μL
Immunoglobulin G	526	861-1,747	mg/dL
Immunoglobulin A	12	93-393	mg/dL
Immunoglobulin M	9	50-269	mg/dL
Total protein	6.0	6.6-8.1	g/dL
Albumin	4.1	4.1-5.1	g/dL
Total bilirubin	0.7	0.4-1.5	mg/dL
Aspartate transaminase	19	13-30	U/L
Alanine transaminase	11	7-23	U/L
Lactate dehydrogenase	242	124-222	U/L
Alkaline phosphatase	112	106-322	U/L
Blood urea nitrogen	12	8-20	mg/dL
Creatinine	0.60	0.46-0.79	mg/dL
Sodium	141	138-145	mmol/L
Pottasium	4.2	3.6-4.8	mmol/L
Chloride	101	101-108	mmol/L
Calcium	9.5	8.8-10.1	mg/dL
Glucose	106	73-109	mg/dL
C-reactive protein	<0.10	<0.14	mg/dL
Anti-aquaporin antibodies	<1.5	<3.0	U/mL
Cerebrospinal fluid			
Opening pressure	110	70-180	mmH_2_O
Cells	1 (mononucleosis cells)	<5	/μL
Protein	34	<50	mg/dL
Glucose	64	50-80	mg/dL
IgG index	0.44	<0.7	
EB virus DNA	<2.0 × 10^2^	<2.0 × 10^2^	copies/mL
HSV DNA	<2.0 × 10^2^	<2.0 × 10^2^	copies/mL
VZV DNA	<2.0 × 10^2^	<2.0 × 10^2^	copies/mL
JCV DNA	1.466 × 10^8^	<2.0 × 10^2^	copies/mL

Cerebrospinal fluid (CSF) examination (Table 1) revealed an opening pressure of 110 mmH_2_O with the appearance of clear fluid containing only mononucleosis cells, protein of 34 mg/dL, glucose of 64 mg/dL, and IgG index of 0.44. CSF examinations for all infections were all negative for: bacterial culture, cryptococcus neoformans antigen, polymerase chain reaction (PCR) for Epstein-Barr Virus deoxyribonucleic acid (DNA), herpes simplex virus DNA, and varicella zoster virus DNA. The PCR of JC virus (JCV) DNA [5,6] in the patient’s CSF was significantly consolidated at 1.466 × 10^8^ copies /mL and confirmed the diagnosis as progressive multifocal leukoencephalopathy (PML). The patient’s spinal cord did not have lesions, as confirmed by spinal MRI. The approach to treatment was thus conservative. The neurological symptoms progressed rapidly, with significant left hemiplegia, dysarthria, and disorder of consciousness. The patient died three months after the onset of neurological symptoms.

**Figure 4:**
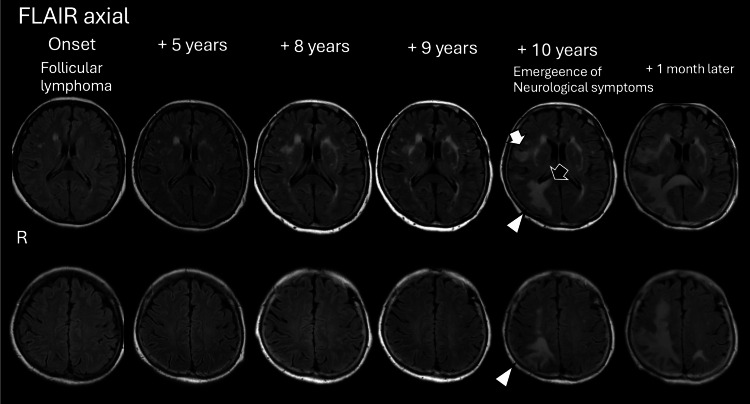
Longitudinal changes of white matter lesions including progressive multifocal leukoencephalopathy (PML) on the brain MRI. Axial FLAIR images are shown from the first onset of follicular lymphoma to PML. When screening brain imaging at FL onset, the patient presented with ischemic patches in the right subcortical white matter and periventricular regions. Due to an incidentally found aneurysm in the left internal carotid artery and clipping at five years after FL onset, a brain MRI was performed annually. At five, eight, and nine years after the onset of FL, ischemic lesions increased. At 10 years from onset, neurological symptoms emerged, and the new lesions of PML seemed to overlap with right ischemic lesions, exhibiting homogeneous quality with swelling (white arrow) and involving the right optic radiation, parietal cortices (arrowhead), and corpus callosum (black arrow). Only a month after the onset of neurological symptoms, the subcortical lesions rapidly widened, and the left side lesion emerged. PML: progressive multifocal leukoencephalopathy; FLAIR: fluid-attenuated inversion recovery; FL: follicular lymphoma; MRI: magnetic resonance imaging.

Brain MRIs obtained from the patient's first episode of FL at the age of her 50s were reviewed (Figure 4, all FLAIR images). Patches of ischemia were observed in the right frontal subcortical regions and the periventricular areas. An incidental finding of an aneurysm was found in the left carotid artery, which had been clipped five years after the onset of FL, prompting monitoring by yearly interval MRI brain scans. Nine years after the onset, the number and area of ischemic lesions had increased, particularly those involving the lateral side of the putamen. With the onset of neurological symptoms, a comparison of the interval MRI images revealed the development of new white matter lesions. There was an overlap between the new PML lesions and existing PML lesions on the right side. The appearance of these white matter lesions was distinctly different, more homogeneous, and expansive compared to the ischemic patches. Some of these lesions spread to the parietal cortices and corpus callosum. The right optic radiation was also involved. These new lesions rapidly progressed through the white matter and even to the left hemisphere within a month after the onset of neurological symptoms.

## Discussion

The patient had three recurrent episodes of FL. The last recurrence was controlled with epcoritamab; however, the complication of JCV infection, which invaded the patient's neurological system, became critical for life. Epcoritamab had an evident effect on FL recurrence. Infection by JCV can lead to the development of PML, which is untreatable. This raises awareness for clinicians of the possible complications of initiating epcoritamab treatment in patients with FL [2].

Epcoritamab is a subcutaneously administered, bispeciﬁc antibody targeting CD3 and CD20 that redirects and activates T cells to kill CD20-expressing malignant cells [3,7]. In a dose-expansion cohort of patients with relapsed or refractory large B-cell lymphoma (LBCL), single-agent epcoritamab demonstrated high overall response rates, including deep and durable complete responses [2]. The adverse events of the therapy were concluded to be manageable and representative of low-grade cytokine release syndrome [2]. The study of epcoritamab for multiple relapsed or refractory FL was ongoing; in the first enrolled group, with a median follow-up of 17.4 months, the overall response rate was 82.0% (95% CI: 74.3-88.3), with a complete response rate of 62.5% (95% CI: 53.5-70.9). The most common grade 3-4 treatment-emergent adverse event was neutropenia in 32 (25%) of 128 patients. Grades 1-2 CRS was reported at 65%, while grade 3 was at 2%. ICANS was reported in six with grades 1 and 2 [2]. To date, the present case has been the first report of a complication of PML during epcoritamab treatment in a serious immunodeficiency state such as lymphocytopenia. In this patient, lymphocytopenia had developed before treatment with epcoritamab. This is probably a general finding in patients with refractory FL. Therefore, we must be cautious about lymphocytopenia and the possibility of infection with JCV when utilizing this novel, effective treatment for refractory FL.

PML is a fatal infection of the central nervous system (CNS) without effective available treatment, caused by reactivation of the JCV and cell-mediated immunodeficiency in the host [8]. This causes the destruction of myelin within subcortical white matter, leading to patchy pattern lesions with diverse neurological symptoms, including changes in mental status, seizures, ataxia, and hemiparesis [8]. Primary infection with JCV typically occurs during childhood, where 50%-90% of the general population has antibodies against JCV, resulting in latent asymptomatic infection in the kidneys and lymphoid tissues. Acquired immunodeficiency syndrome (AIDS), organ transplantation, or immunosuppressive treatments reactivate JCV and cause PML. Recently, disease-modifying therapies (DMTs) for multiple sclerosis (MS) are known to increase risk [9].

Consensus statement proposals in the PML section of the American Academy of Neuroinfectious Diseases suggest that the diagnosis of JCV should be guided by clinical features, compatible radiological findings, and CSF PCR [1]. In addition, the distribution of subcortical lesions and characteristic intensity patterns provide clues to the diagnosis of PML for a longer period of monitoring with yearly interval brain MRI scans after the onset of FL, as in the present patient. The patient incidentally had an aneurysm, which induced regular radiological follow-up approximately five years before the diagnosis of PML. Although patchy white matter lesions of PML are usually difficult to differentiate from chronic ischemic patches, brain MRI imaging taken before the development of PML can be compared with images taken after the onset of PML. The patient had subcortical lesions five, eight, and nine years after the onset of FL, which were not expanded in size in white matter in contrast to that in PML, often expressed as "swelling" (Figure 4, white arrow). The invasion of white matter with homogeneous quality (Figure 4, arrowhead) reaching the corpus callosum (Figure 4, black arrow) is also a characteristic finding of PML, and these observations could allow for comparison with appearances before the onset of PML.

An increasing risk of PML has been reported in patients receiving monoclonal therapy and other drugs. The highest incidence of PML occurs with natalizumab, a monoclonal antibody (mAb) against alpha-4 integrin as a representative of DMT for MS, with an overall global incidence of 3.94 per 1000 patients [9]. Fingolimod, a sphingosine-1-phosphate receptor (S1PR) modulator for MS, has an incidence of 0.069 per 1,000 patient-years [4]. This appears to have a higher incidence in comparison to those HIV-positive patients of 1.3 per 1,000 patient-years from 2000 to 2006 in Denmark [10]. Among patients exposed to rituximab, a monoclonal anti-CD20 antibody drug, the incidence rate of PML was 2.89 per 1,000 patient-years in the subgroup of non-Hodgkin disease [11,12]. The case fatality rate was reported to be up to 90%, and the median time to death after PML diagnosis was 2.0 months [13]. Three patients with FL included in the study had a CD4-positive lymphocyte count of 174-570/μL at the onset of PML, whereas those of the other diseases ranged from 68 to 2,100/μL [13]. Historically, PML has been known as a complication among HIV-positive patients [8]; however, awareness should be focused on immune-mediated therapies developing among rheumatoid, neuroimmunological, and hematological malignant diseases. Patients with FL are considered at risk of severe infection after rituximab treatment [14], where CD4-positive lymphocyte counts are usually decreased by lymphoma recurrence, similar to the present case.

To defend against CNS infections, the trafficking of immune cells from the periphery to the CNS is essential. Drugs like natalizumab can obstruct leucocyte migration that induces PML [15]. Anti-JCV antibody testing [16] is not standardized, except for natalizumab-treated patients. However, it is recommended to test every six months for anti-JCV antibody-negative patients or antibody-positive patients with index levels ≤1.5. For those with an anti-JCV antibody index >1.5, further testing is not mandatory [17]. The association between high viral loads in CSF at the time of diagnosis and poorer outcomes has been reported in several studies [15]. Reuwer et al. emphasized the importance of early diagnosis and intervention when the viral load is still low. The interventions were listed as plasmapheresis, methylprednisolone, mirtazapine, and mefloquine in various combinations, but there have still been no evident effects on PML with even MS patients' studies [15]. For patients without MS, as in the present case, we hope to develop the utility of anti-JCV antibody testing with diverse medications, including epcoritamab, that can change the immune system.

## Conclusions

A patient diagnosed with PML showed the potential danger of JCV infection through their characteristic subcortical lesions and various neurological symptoms. The new drug epcoritamab, a bispecific antibody targeting CD3 and CD20 that redirects and activates T cells for the treatment of FL in the present case, exerted a fair effect on lymphomas. However, it emphasized lymphocytopenia in recurrent FL that led to JCV proliferation in the central nervous system.

When targeting hematological malignancies and neuroinflammatory and rheumatological diseases with antibody drugs, JCV and PML should be given priority consideration. Detecting JCV in the brain as small subcortical U-fiber lesions and recognizing associated symptoms is crucial for early diagnosis.
